# Shaping the future of liver surgery: Implementation of experimental insights into liver regeneration

**DOI:** 10.1007/s10353-018-0515-3

**Published:** 2018-03-06

**Authors:** D. Pereyra, P. Starlinger

**Affiliations:** Department of Surgery, General Hospital, Medical University of Vienna, Währinger Gürtel 18–20, 1090 Vienna, Austria

**Keywords:** Postoperative Outcome, Liver Dysfunction, Therapeutic Approaches, Hepatic Progenitor Cells, Von Willebrand Factor

## Abstract

**Background:**

While liver surgery has become a safe and feasible operation technique, the incidence of postoperative liver dysfunction still remains a central problem. Approximately 10% of patients undergoing liver resection were shown to develop liver dysfunction, which is associated with an increased risk of morbidity and mortality. Yet, to date there is no effective treatment option for postoperative liver dysfunction available. The development of postoperative liver dysfunction was linked to a disruption in the liver’s potential to regenerate. Thus, it is importance to elucidate the underlying mechanisms of liver regeneration and to find potential therapeutic targets for the treatment of patients with postoperative liver dysfunction.

**Methods:**

A review of the literature was carried out.

**Results:**

We report on potential future interventions for improvement of liver regeneration after surgical resection. Moreover, we evaluate the benefits and drawbacks of hepatic progenitor cell therapy and hematopoietic stem cell therapy. However, the most significant improvement seems to come from molecular targets. Indeed, von Willebrand factor and its pharmacologic manipulation are among the most promising therapeutic targets to date. Furthermore, using the example of platelet-based therapy, we stress the potentially adverse effects of treatments for postoperative liver dysfunction.

**Conclusion:**

The present review reports on the newest advances in the field of regenerative science, but also underlines the need for more research in the field of postoperative liver regeneration, especially in regard to translational studies.

## Introduction

Today, liver resection represents a safe and feasible therapeutic option for the treatment of malignant and benign liver tumors [1, 2]. This is due to a continuous refinement of surgical strategies, starting from a simple left lobe resection in 1888 and evolving into an adjustable procedure with a high degree of personalization to the patients’ needs [3]. The former limitations of liver surgery, namely, bleeding at the site of resection, bilobar tumor growth, and multiple metastasis within the liver, could be overcome, which makes major resection of up to 75% of the livers initial volume a feasible and safe operation [4, 5]. Indeed, postoperative mortality is reported to affect no more than 2–8% of patients undergoing liver resection, with the latter percentage referring to high-risk patients who require complex resections [6–9]. Of note, this is also due to advances in perioperative management of patients undergoing liver resection. In addition, the preoperative optimization of patients needing extensive liver resection has improved, with the most prominent example being preoperative portal vein embolization [10]. This technique allows for major resection also in patients with an initial future liver remnant of less than 25%, as it triggers regeneration in the healthy liver parenchyma.

**Fig. 1:**
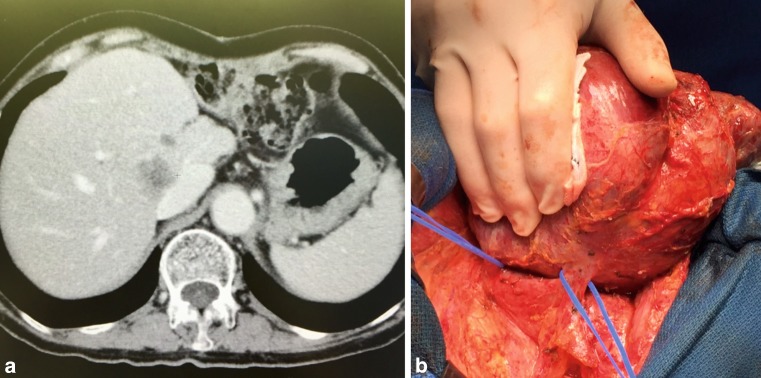
Repeated liver resections are possible due to well-functioning liver regeneration*.* **a** Computer tomography scan of a patient with recurrence of metastatic disease in the liver after a precedent liver resection. **b** Still, potentially curative liver resection could be performed, as the patient’s liver fully regenerated in volume and function

While mortality rates in this specific patient cohort are within acceptable ranges, postoperative morbidity and prolonged hospitalization, including the need for intensive care unit management and specific interventions, still remain an important concern. According to recent literature, the incidence of postoperative morbidity remains stable at 30–50% [6, 9]. Interestingly, postoperative complications have been associated with the development of liver dysfunction (LD) and delayed hepatic regeneration [11]. Indeed, a well-functioning liver regeneration after resection is believed to be the central determinant of clinical outcome [12]. Further, a well-functioning liver regeneration allows for multiple consecutive resections if needed (Fig. 1). However, if the regenerative capacity is impaired and a patient develops postoperative LD, the clinical consequences are inevitable because to date no therapeutic approach to support postoperative liver regeneration has been identified. The fact that more than 65% of patients with postoperative LD will develop major complications highlights the need for adequate treatment options to support postoperative liver regeneration [13]. In the present review, we aim to present an overview of the most promising experimental data regarding potential therapeutic targets to support liver regeneration in patients suffering from postoperative LD.

## Hepatic progenitor cells and cellular therapy

The liver consists of a variety of different cell types that display a high degree of interaction and niche dependency. Beside the main parenchymal cells, which are hepatocytes, liver-specific endothelial cells, cholangiocytes, hepatic stellate cells, and stationary macrophages (i. e., Kupffer cells) are the most frequent cell types in the liver [14]. These cells all show a high level of differentiation and specialization, which is also visible when looking at the different functions they need to fulfil. Recently, oval cells have been described as a potential hepatic progenitor cell (HPC) niche [15]. Indeed, this cell type was shown to have the ability to give rise to both hepatocytes and cholangiocytes, which led to the hypothesis that oval cells might be relevant for liver regeneration after tissue damage. Moreover, inducible progenitors were shown to be crucial for liver regeneration, and inhibition of their activation in a mouse model led to impaired liver regeneration after partial hepatectomy [16]. Thus, activation of HPCs and enforcement of differentiation to mature hepatocytes might be a potential therapeutic target for induction of liver regeneration. Español-Suñer et al. were able to show that in vivo activation of oval cells can be performed via the PGI_2_ analogue iloprost [17]. Although the arising hepatocytes showed a typical behavior and were able to proliferate upon activating stimuli in vivo, these cells only contributed to 2.45% of the total hepatocytes. Further, Español-Suñer and coworkers did not observe a massive increase in oval cell activation after partial hepatectomy in mice.

Interestingly, HPCs were found to be regulated by, if not even derived from, hematopoietic stem cells (HSC). These cells share a large panel of surface markers [18]. Furthermore, Y chromosomes were detected in female patients’ hepatocytes after bone marrow transplantation from a male donor [19]. Thus, a plethora of experimental studies addressed the question of whether cellular therapy via HSCs might contribute to liver regeneration in different models [20]. In conclusion, the studies provide diverging results: While HSCs seemed to improve the outcome of mice after liver damage, this improvement could not consistently be traced back to the engraftment of HSCs [21]. In some studies, an engraftment was only observed for 4% of transplanted HSCs. Nevertheless, a compensatory proliferation of hepatocytes took place in mice treated with HSCs. Accordingly, the beneficial effects of HSC therapy were thought to be either dependent on their ability to scavenge and neutralize reactive oxygen species or due to paracrine effects on hepatocytes [22, 23].

Taken together, both HPCs and HSCs might have a beneficial effect on liver regeneration. However, the available data are not conclusive yet and translational data as well as prospective human trials are needed to clarify the clinical applicability of this kind of cellular therapy.

## Molecular targets—von Willebrand factor

In the past few years, molecular targets became more innovative in the field of liver regeneration. This might be due to the increased interest in pathophysiologic processes taking place during the complex process of liver regeneration. However, while there are abundant data on rodent models and in vitro experiments, only few translational data have been obtained to date. In this context, we recently published a translational study focusing on von Willebrand factor antigen (vWF-Ag) during postoperative liver regeneration in humans [24]. VWF-Ag is known for its role in primary hemostasis, where it facilitates the accumulation of platelets and their binding to the sub-endothelial matrix [25]. It is stored both in granules of platelets and endothelial cells and can be released upon stimulation. Previously, vWF-Ag was proposed as a marker for portal hypertension and cirrhosis [26]. However, the role of vWF-Ag in patients undergoing liver resection has not been addressed before. While a large clinical study showed that patients with high preoperative levels of vWF-Ag had a significantly increased risk for development of postoperative LD, complications, and even mortality, we aimed to provide more insight into the pathophysiological involvement of vWF-Ag in liver regeneration. Interestingly, Kirschbaum et al. recently published experimental data on the role of vWF-Ag during liver regeneration in mice [27]. They showed the central importance of vWF-Ag-dependent platelet accumulation especially during the early period after induction of liver regeneration. Indeed, the significance of platelets and platelet stored factors for a well-functioning liver regeneration has previously been shown by our group as well as by other researchers [12, 28, 29]. Thus, we took a closer look at vWF-Ag secretion during the early phase of liver regeneration (i. e., the first 2 h after portal vein ligation) in the human setting. Astonishingly, our results precisely followed the experimental data by Kirschbaum et al. We were able to validate previous reports on platelet accumulation within the regenerating liver. Interestingly, we also observed an increase in vWF-Ag within no more than 2 h of induction of liver regeneration. Most importantly, this initial increase was only seen in patients who did not develop LD in the postoperative time course, while patients with LD were not able to substantially secrete vWF-Ag in the early phase of liver regeneration. This led to the hypothesis that an initial burst in vWF-Ag might be relevant for induction of platelet-mediated liver regeneration.

**Fig. 2:**
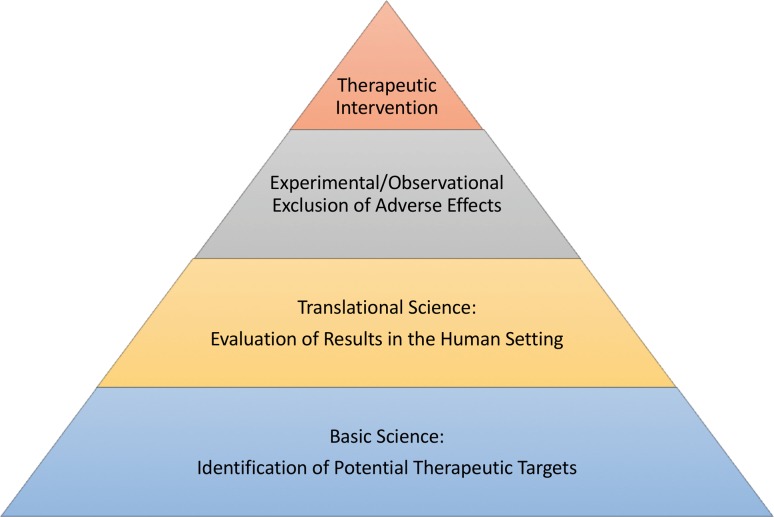
Scheme of research construction in the field of liver regeneration*.* While basic science is clearly the basis of good research in liver regeneration, as it is vital for identification of potential therapeutic targets, translational research, comprising validation of basic research results and evaluation in the human setting, is of central importance. However, potential interfering factors as well as of potentially adverse effects need to be excluded before an evaluated target can be considered trustworthy for any kind of therapeutic intervention

Overall, this study provides an example of how therapeutic targets in the field of liver regeneration can be identified (for a summary, refer to Fig. 2). Thinking one step further, these data would suggest a beneficial effect of pharmacologic manipulation of vWF-Ag secretion. Indeed, drugs inducing this effect, such as vasopressin analogues, are already part of clinical routine. Moreover, a study investigating the use of terlipressin in patients undergoing major liver resection is currently being conducted (ClinicalTrials.gov Identifier: NCT01921985). Thus, this intervention might be the first treatment option for patients suffering from postoperative LD and might concomitantly improve the overall outcome of patients undergoing liver resection.

## Drawbacks from treatments

While several different targets to treat patients suffering from postoperative LD are being investigated, not all of them will ultimately find their way into clinical practice. This can either be due to a reduced effectiveness in the human setting, as might be the case for HPC/HSC-based therapy, or that potential adverse effects of the respective treatment need to be investigated. Indeed, we and others previously hypothesized a potential positive effect of platelet-based therapy on the outcome of liver regeneration [12, 28, 29]. Specifically, within a previous study we showed that patients with low preoperative levels of intra-platelet serotonin (IP-5HT) prior to the operation have a higher risk for development of postoperative LD [30]. Thus, a pharmacological elevation of IP-5HT seemed to be a reasonable treatment option in patients undergoing liver resection. However, 5HT was also proposed as a pro-proliferative molecule for various malignant diseases including all main tumor entities of the liver [31–34]. Hence, we conducted a study with the aim to elucidate the influence of IP-5HT on the oncological outcome of patients undergoing liver resection [35]. Strikingly, patients with high preoperative levels of IP-5HT were found to suffer more frequently from early tumor recurrence within 1 year postoperatively. This study put a huge caveat on platelet-based therapy. The initially considered pharmacological manipulation might induce adverse effects and a potential intervention has to be investigated with caution. Likewise, not only 5HT, but also other platelet-contained factors were shown to interact with malignant cells and metastasis [36]. Further, these data question the beneficial effect of a proliferation-based approach for improvement of postoperative liver regeneration, as induction of proliferation in hepatocytes most likely also induces proliferation in remaining micro-metastases and circulating tumor cells.

## Conclusion

Liver regeneration is a highly complex process. Yet, its understanding is of pivotal importance to improve the outcome of patients undergoing liver resection. While a multitude of potential therapeutic targets were established in the past (most important results are summarized in Table 1), only few have the potential to ultimately act as a promising therapy for patients suffering from postoperative LD. Hence, more research, especially in the translational field, is imperatively needed. However, future research should not focus exclusively on basic science, as artificial models often neglect important aspects of the multifactorial process of liver regeneration and cancer and thus might overestimate certain results.

**Table 1:** Summary of the results

Section	Examples	Results
Cell-based therapy	Hepatic progenitor cells Hematopoietic stem cells	Good results in experimental models Clinical application needs to be evaluated
Molecular targets	von Willebrand factor	Experimental evaluation showed good results Translational data available Pharmacologic intervention in humans currently under investigation
Drawbacks	Platelets/serotonin	Central for liver regeneration and thus a potential target for improvement of liver regeneration However, also pro-proliferative for malignant cells and an association with early disease recurrence was shown

## Abbreviations

HPC: Hepatic progenitor cell
HSC: Hematopoietic stem cells
IP-5HT: Intra-platelet serotonin
LD: Liver dysfunction
vWF-Ag: von Willebrand factor antigen
